# Blue Light Sensing in *Listeria monocytogenes* Is Temperature-Dependent and the Transcriptional Response to It Is Predominantly SigB-Dependent

**DOI:** 10.3389/fmicb.2019.02497

**Published:** 2019-11-14

**Authors:** Amber L. Dorey, Bo-Hyung Lee, Bjorn Rotter, Conor P. O’Byrne

**Affiliations:** ^1^Microbiology, School of Natural Sciences, Bacterial Stress Response Group, National University of Ireland Galway, Galway, Ireland; ^2^GenXPro, Frankfurt am Main, Germany

**Keywords:** *Listeria monocytogenes*, blue light, SigB, temperature, transcriptional responses, RNA seq, RsbL, Lmo0799

## Abstract

*Listeria monocytogenes* is an important food-borne pathogen that is tolerant to many of the stresses commonly used during food preservation. Outside the host, the bacterium has a saprophytic lifestyle that includes periodic exposure to solar irradiance. The blue component of this light is known to influence the activity of the stress-inducible sigma factor Sigma B (σ^B^). In this study, the influence of temperature and growth phase on the response of *L. monocytogenes* to blue light was investigated and the global transcriptional response to blue light was elucidated using an RNAseq-based approach. Stationary phase cells were found to be significantly more resistant to killing by blue light (470 nm) than exponential phase cells. Temperature also had a marked effect on blue light resistance with cells cultured at 37°C being much more sensitive than cells grown at 30°C. The role of σ^B^ in light tolerance was confirmed but this effect was observed only at 30°C. σ^B^ activation by blue light was assessed by measuring the transcriptional response of known σ^B^-dependent genes (*sigB*, *lmo2230*, and *opuCA*) to light. The transcripts were induced by blue light only at 30°C suggesting that blue light fails to activate σ^B^ at 37°C. The light-induced transcription at 30°C was dependent on a functional blue light sensor, Lmo0799 (which we rename herein as RsbL). A transcriptomic analysis of the response to sub-lethal levels of blue light found that the changes in transcription were almost entirely σ^B^-dependent. A mutant where the light sensing mechanism of RsbL was inactivated through an amino acid substitution (Cys56Ala) was found to have an attenuated response to blue light, but residual activation of σ^B^-dependent genes suggested that alternative routes for activation of σ^B^ by light are likely to exist. Overall, the study highlights the central role of σ^B^ in the response of this pathogen to visible light and further shows that light sensing is absent at temperatures that exist within the mammalian host.

## Introduction

The Gram positive rod-shaped bacterium *Listeria monocytogenes* is a ubiquitous organism in the environment and can cause infection when it enters the host *via* the food chain. In order to survive, the bacteria must be able to sense and respond to various environmental stimuli, including light. Photodynamic inactivation (PDI) of *L. monocytogenes* by visible light has been shown to be an effective bactericidal mechanism (Maclean et al., 2009; Endarko et al., 2012), which could potentially be utilized in food processing environments as an adjunct to existing food preservation measures. However, the factors that influence sensitivity to visible light are not well understood and thus this study sought to investigate how the culture conditions and the presence of environmental stressors might influence susceptibility to PDI.

The resistance of *L. monocytogenes* to environmental stresses has been partly attributed to the alternative sigma factor σ^B^ (Ferreira et al., 2003; Chaturongakul and Boor, 2006; Utratna et al., 2011). The stressosome protein complex was first discovered in *Bacillus subtilis* (Kim et al., 2004), and it is required to detect environmental stress signals and initiate the signaling cascade required to activate σ^B^ (Marles-Wright et al., 2008). In *L. monocytogenes*, the stressosome is composed of a core of RsbS and RsbT proteins, with RsbR and its paralogue proteins embedded into this core (Impens et al., 2017). The blue light sensor protein Lmo0799 (herein renamed RsbL) is an RsbR paralogue containing a light-oxygen-voltage (LOV) N terminal domain (Ondrusch and Kreft, 2011; Impens et al., 2017); however, the sensory functions of the other four paralogues are currently unknown. As in *B. subtilis* (Gaidenko et al., 2006), the exposure of *L. monocytogenes* to visible light activates σ^B^ (Ondrusch and Kreft, 2011), which is required to induce transcription of the general stress response regulon (O’Byrne and Karatzas, 2008). The fortuitous discovery that oscillating cycles of light and dark results in a ringed colony morphology in *L. monocytogenes* further confirmed that RsbL is required for σ^B^ activation by light (Tiensuu et al., 2013). In the absence of either RsbL or σ^B^, *L. monocytogenes* is unable to form the ringed colony morphology during the oscillating cycles of light and dark (Tiensuu et al., 2013).

Several amino acids are conserved between YtvA (the *B. subtilis* homologue) and RsbL, including the cysteine residue at positions 62 and 56 in YtvA and RsbL, respectively, that is required for the formation of a photoadduct with the flavin mononucleotide cofactor in response to blue light (Gaidenko et al., 2006; O’Donoghue et al., 2016). In both the deletion mutant Δ*rsbL* and the missense mutant *rsbL*-C56A, where this critical cysteine residue is mutated to an alanine, several phenotypes associated with the exposure of *L. monocytogenes* to blue light are abolished (O’Donoghue et al., 2016). These phenotypes include the inhibition of motility (Ondrusch and Kreft, 2011), a ringed colony morphology in the presence of oscillating cycles of light and dark (Tiensuu et al., 2013), and inhibited growth in the presence of low doses of blue light (O’Donoghue et al., 2016). Therefore, the presence of this conserved cysteine residue is required to sense light in *L. monocytogenes*, and the mutant lacking it effectively behaves as a blind strain in respect to blue light.

Temperature is an important environmental parameter that influences the physiology and behavior of *L. monocytogenes* in a variety of ways. Certain stresses that *L. monocytogenes* is exposed to during food preservation, such as osmotic and acid stress, are also encountered within the host, and the effect of temperature on the ability of *L. monocytogenes* to adapt to these stresses has been studied (Bergholz et al., 2012; Shen et al., 2014). When changes in gene transcription in response to osmotic stress at both 7 and 37°C were measured, 888 genes showed altered transcriptional profiles between the two temperatures (Bergholz et al., 2012). This set of genes included *sigB* and several other σ^B^-dependent genes involved in adaptation to stress conditions and pathogenesis (Bergholz et al., 2012). Similarly, it has been demonstrated that, while *L. monocytogenes* can display an adaptive response to acid stress at 30°C (Davis et al., 1996), cells are unable to show the same adaptation at 4°C (Shen et al., 2014).

It has been known for over 30 years that *L. monocytogenes* only expresses flagella at temperatures below 37°C (Peel et al., 1988); however, a Δ*sigB* deletion mutant has increased motility at 37°C compared to the wild-type (Raengpradub et al., 2008). The transcriptomic study by Toledo-Arana et al. (2009) identified a σ^B^ promoter upstream of *mogR*, a transcriptional repressor, that enables the transcription of three genes involved in flagellar biosynthesis, *lmo0675*, *fliP*, and *fliQ*. Although this transcript is over-expressed in stationary phase, transcription is unaffected by temperature (Toledo-Arana et al., 2009). Therefore, growth temperature is an important variable, whose influence on the physiology of *L. monocytogenes* could impact the susceptibility of this pathogen to PDI.

Studies concerning several bacterial species, including *L. monocytogenes*, and their killing by light have been conducted. The sensitivity of *Staphylococcus aureus* to killing by visible light is highly dependent upon the environmental oxygen concentrations, with sensitivity increasing as oxygen levels increase (Maclean et al., 2008). A comparison between *Salmonella enteritidis*, *Escherichia coli*, and *Campylobacter jejuni* demonstrated that while all three species could be killed by visible light, *C. jejuni* showed a significantly increased sensitivity to killing compared to the other two species (Murdoch et al., 2010). In a similar study comparing the sensitivity of *L. monocytogenes*, *S. enterica*, *S. sonnei*, and *E. coli* to visible light, *L. monocytogenes* was significantly more sensitive than the other species in liquid culture, but the results varied on solid surfaces (Murdoch et al., 2012). Taken together, the results of these studies suggest that visible light may have the potential to be an effective antimicrobial; however, the response of bacteria to visible light and the influence of environmental factors on this response need to be understood in order to optimize the use of visible light as control measure.

While a role for σ^B^ in resistance to killing by visible light has been shown, somewhat surprisingly neither the Δ*rsbL* nor the *rsbL*-C56A mutants show a change in sensitivity to killing by visible light (O’Donoghue et al., 2016). However, increased transcription of the σ^B^-dependent gene *lmo2230* in response to visible light requires RsbL (Tiensuu et al., 2013), suggesting that *L. monocytogenes* may respond to blue light *via* an RsbL-independent mechanism. Despite evidence that visible light may be an effective antimicrobial, very little is known about the global transcriptomic response of *L. monocytogenes* to visible light, and indeed this is true of most non-phototrophic bacteria. In the present study, we also sought to investigate the influence of environmental stressors that could be present in a food processing environment on light tolerance and also to elucidate the transcriptional response of *L. monocytogenes* to visible light *via* whole transcriptomic analysis in order to develop a deeper understanding of how this important food pathogen senses and responds to visible light.

## Materials and Methods

### Bacterial Strains and Culture Conditions

The bacterial strains used in this study are listed in Table 1. Permanent stocks of each strain were stored in brain heart infusion (BHI) (LabM) broth supplemented with 7% (v/v) dimethyl sulfoxide (Sigma-Aldrich) at −80°C. Permanent stocks were streaked onto agar, incubated at 37°C overnight then stored at 4°C for up to 1 month. For overnight cultures, several colonies were taken from an agar plate, inoculated into BHI broth, and incubated at either 30 or 37°C, shaking, in darkness. For experiments requiring exponentially growing cells, overnight cultures were diluted to OD_600_ 0.05 in BHI broth and incubated at either 30 or 37°C, shaking, in darkness until OD_600_ ~ 0.2.

**Table 1:** Bacterial strains used in this study.

COB strain number	Strain	Genotype	Source
*261*	*Listeria monocytogenes*	*L. monocytogenes* EGDe wild-type	Cormac Gahan
*262*	*Listeria monocytogenes*	*L. monocytogenes* EGDe Δ*sigB*	Cormac Gahan
*610*	*Listeria monocytogenes*	*L. monocytogenes* EGDe with C56A replacement at *lmo0799* gene strain A	Beth O’Donoghue

### Growth Phase-Dependent Light Survival Assay

Stationary and exponential phase cells (OD_600_ ~ 0.2) grown at 37°C were centrifuged at 9,000 × *g* for 5 min at room temperature, washed once in phosphate buffered saline (PBS) (Sigma-Aldrich), and resuspended in PBS to OD_600_ 1 (~10^9^ cfu/ml). Two hundred microliter aliquots were made in triplicate in two round-bottomed 96-well plates (ThermoFisher) and incubated for 8 h at 30°C, with one plate exposed to 35 mW cm^−2^ blue (470 nm) light and the other wrapped in aluminium foil. At 2 h intervals, samples were taken for each strain, diluted to 10^−7^ in PBS, and 10 μl per dilution was plated in triplicate onto BHI agar plates. Plates were incubated at 37°C for 48 h in darkness and cfu/ml was calculated. The average results from two biological replicates are shown with SD.

### Growth Temperature-Dependent Light Survival Assay

Exponential phase cells (OD_600_ ~ 0.2) grown at 30 and 37°C were centrifuged at 9,000 × *g* for 5 min at room temperature, washed once in phosphate buffered saline (PBS) (Sigma-Aldrich), and resuspended in PBS to OD_600_ 1 (10^9^ cfu/ml). Two hundred microliter aliquots were made in triplicate in two round-bottomed 96-well plates (ThermoFisher) and incubated for 6 h at 30°C, with one plate exposed to 35 mW cm^−2^ blue (470 nm) light and the other wrapped in aluminium foil. At 0 and 6 h, samples were taken for each strain, diluted to 10^−7^ in PBS, and 10 μl per dilution was plated in triplicate onto BHI agar plates. Plates were incubated at 37°C for 48 h in darkness and cfu/ml was calculated. The average results from two biological replicates are shown with SD.

### RNA Sample Collection and Isolation

For RNA sample collection, exponentially growing cultures at 30 and 37°C were divided into two, with one exposed to 0.6 mW cm^−2^ and one in darkness, and kept at the same temperature at which they had been growing. Samples were collected at 10 min intervals and stabilized in RNALater (Sigma). RNA was extracted using the RNeasy mini kit (Qiagen) according to manufacturers’ instructions. Cells were lysed by bead beating for two 40 s cycles at 6 m/s in FastPrep® 1 ml Matrix B lysis tubes (MP Biomedicals) with a FastPrep®-24 *Classic* Instrument (MP Biomedicals). The RNA was quantified by NanoDrop and contaminating DNA was removed using TURBO DNA-free (Ambion) according to manufacturers’ instructions. RNA was quantified and the RNA Integrity was measured using the Bioanalyzer (Agilent) according to manufacturers’ instructions. Only RNA with a RIN of greater than 8 was accepted for conversion to cDNA. For cDNA generation, 15 μl of RNA was added to 1 μl 10 mM dNTPs (Sigma) and 1 μl random primers (Invitrogen) and incubated 5 min and 65°C then on ice for 1 min. The sample was centrifuged 5 s and 4 μl first strand buffer, 1 μl 0.1 M DTT, and 1 μl Superscript III (all Invitrogen) was added. The sample was incubated for 5 min at 25°C, 60 min at 50°C, and 15 min at 70°C and then stored immediately at −80°C.

### Real-Time PCR

RT-PCR was carried out using the LightCycler® 480 (Roche) using a total volume of 10 μl. All primer sequences, and their corresponding efficiencies, used in this study are listed in Table 2, and reactions were run using 2X QuantiTect SYBR Green (Qiagen). Primer efficiencies were calculated using gDNA diluted 10-fold to 10^−5^ and analyzed using the LightCycler® 480 software 1.5 (Roche). For all samples, a minimum of two biological replicates were performed in triplicate. The housekeeping gene 16 S was used as an internal standard (Tasara et al., 2007). Sample Cp values were calculated using LightCycler 480 software 1.5, and relative expression was calculated using Microsoft Excel by the method described by Pfaffl (2001). Statistical significance was determined by paired *t*-test or two-way ANOVA with Dunnett’s multiple comparisons test. Statistical significance was determined between strains at each time point.

**Table 2:** RT-PCR primers and their corresponding efficiencies used in this study.

COB primer number	Primer sequence	Primer name	Primer efficiency	Origin
625	CTATATTTGGATTGCCGCTTAC	sigB-F RT-PCR	1.95	Marta Utratna
626	CAAACGTTGCATCATATCTTC	sigB-R RT-PCR		Marta Utratna
627	CATCGATAAAGGAGAATTTG	opuCA-F RT-PCR	1.77	Marta Utratna
628	CATAACCAATTGAGCGTCTTAG	opuCA-R RT-PCR		Marta Utratna
629	CATATTCGAAGTGCCATTGC	lmo2230-F RT-PCR	2.00	Marta Utratna
630	CTGAACTAGGTGAATAAGACAAAC	lmo2230-R RT-PCR		Marta Utratna
672	TGGGGAGCAAACAGGATTAG	16S-F RT PCR for 16S RNA	1.95	Marta Utratna
673	TAAGGTTCTTCGCGTTGCTT	16S-R RT PCR for 16S RNA		Marta Utratna
891	TTTGGCGAAATTCCGGTGATGA	lmo0799 FWD RT-PCR	1.83	This Study
892	AACACACGACCGTTTTCAGCA	lmo0799 REV RT-PCR		This Study

### RNA Processing for RNA seq by GenXPro

Upon arrival at GenXPro, the three biological replicates of RNA was stored at −80°C until analysis by GenXPro, as described by Feil et al. (2017). The RIN was assessed again by Labchip GX II Bioanalyzer (Perkin Elmer), and DNA contamination was removed using Baseline-Zero DNase (Epicenter) following the GenXpro in-house protocol. The RNA was incubated in the presence of Baseline-Zero DNase and RiboLock RNase inhibitor (Thermo Fisher Scientific) for 30 min at 37°C. Two volumes of RNA binding buffer (Thermo Fisher Scientific) and absolute ethanol (Roth) were added to the sample, and the mixture was transferred to a Zymo-Spin™ IC Column (Zymo Research). The column was centrifuged 12,000 × *g* for 30 s, and the bound RNA was washed twice with RNA Wash Buffer (Zymo Research) and eluted in nuclease-free water (Zymo Research) as per the manufacturer’s protocol. RNA was re-quantified *via* the fluorescence-based Qubit™ RNA HS assay (Thermo Fisher Scientific) following the manufacturer’s protocol.

Total RNA was treated with Ribo-Zero rRNA removal kit (Illumina) to remove rRNA and enrich mRNA following the manufacturer’s protocol. Briefly, the magnetic beads were washed twice with nuclease-free water and resuspended in Magnetic Bead Resuspension Solution and RiboGuard RNase Inhibitor. To hybridize the beads with the probes, RNase-free water, Ribo-Zero Reaction Buffer, and Ribo-Zero Removal Solution were added to the beads and heated to 68°C for 10 min. Five hundred nanogram total RNA was added to the mixture and incubated at room temperature for 5 min and then 50°C for 5 min. The tube was placed on a magnetic stand, and the depleted RNA was removed in the supernatant and transferred to a separate tube. The enriched mRNA was purified using a Zymo-Spin Column (Zymo Research), and the eluted mRNA was checked for rRNA contamination by Labchip GX II Bioanalyzer.

The NEBNext® Ultra™ II Directional RNA Library Prep Kit for Illumina (Illumina) was used to prepare the cDNA fragment libraries. The enriched mRNA was incubated for 15 min at 94°C to fragment into pieces ~200 nt. NEBNext Strand Specificity Reagent and NEBNext First Strand Synthesis Enzyme Mix were added to the reaction. To enable reverse transcription for first-strand cDNA synthesis, the mixture was heated for 10 min at 25°C, 15 min at 42°C, and 15 min at 70°C, before cooling to 4°C. NEBNext Second Strand Synthesis Reaction Buffer with dUTP Mix (10X), NEBNext Second Strand Synthesis Enzyme Mix, and nuclease-free water were added to the mixture, and the mixture was heated for 1 h at 16°C to enable second-strand cDNA synthesis. The ds cDNA was purified using NucleoMag® NGS Clean-up and Size Select (Machery-Nagel). Briefly, the sample was mixed with NucleoMag® NGS beads in a 1:1 ratio, incubated at room temperature for 5 min, and then the beads were separated from the supernatant using a NucleoMag® SEP magnetic separator for 5 min. The supernatant was removed and discarded. Beads were washed twice with 80% ethanol and dried by incubating at room temperature for 5–15 min. End repair was performed on the ds cDNA library followed by ligation of adaptors, and the purified DNA fragment library was eluted in elution buffer. To determine appropriate cycle numbers for selective enrichment of library fragments by high fidelity PCR, qRT-PCR (Applied Biosystems) was performed using KAPA Hifi polymerase (Roche) with EvaGreen® (Biotium). NEBNext Multiplex Oligos for Illumina (Dual Index Primers) and KAPA Hifi polymerase was used for selective enrichment by high fidelity PCR. PCR products were purified twice using NucleoMag SPRI beads, and the quality of the final library was assessed on Labchip GX II Bioanalyzer. Indexed and purified libraries were loaded together onto a flow cell, and sequencing was carried out on the Illumina NextSeq 500 platform (paired-end, 2 × 75 bp per read).

Sequencing quality was assessed using FastQC and Illumina adapter sequences, and low-quality base pairs were removed using CutAdapt version 1.9 (Martin, 2011). Reads were mapped to the complete sequenced genome of reference strains EGDe (ENSEMBL ASM19603v1) using Bowtie 2 v 2.2.4 with standard parameters and sensitive-local (Langmead and Salzberg, 2012). BAM alignment files were used as input for read counting using htseq-count (HTSeq version 0.6.0) (Anders et al., 2015). Differential expression analyses were performed using DESeq2 in R v 3.2.2 (Love et al., 2014). The differential expression was reported as log_2_ fold changes, with *p* adjusted by the DESeq2 default Benjamini-Hochberg (BH) adjustment method and genes with a > 2-fold change in expression and a *p* < 0.05 was considered as DE.

### Protein Sample Preparation

For all protein samples, 25 ml was taken from stationary phase cultures, and 10 μg ml^−1^ was added before protein extraction to stop protein translation. Samples were centrifuged for 15 min at 9,000 × *g* at 4°C. The bacterial pellet was resuspended in 2 ml sonication buffer [10 mM Tris–HCl (Sigma Aldrich), 0.1 mM EDTA (AnalaR), 5 mM MgCl_2_ (AnalaR), adjusted to pH 8 and autoclaved] supplemented with 2 mg/ml lysozyme (Sigma-Aldrich) and incubated for 30 min at 37°C, shaking. The culture was centrifuged 9,000 × *g* for 15 min at 4°C, and the pellet was resuspended in 0.5 ml of sonication buffer supplemented with 1% (vol/vol) protease inhibitor (Sigma-Aldrich). The culture was transferred to a 2 ml screw cap tube containing 0.25 ml 0.50 mm and 0.50 ml 0.10 mm zirconia beads (Thistle Scientific) and vortexed for 10 min, alternating 30 s bead beating and 30 s rest on ice. The preparation was centrifuged 13,000 × *g* for 30 min at 4°C to remove cell debris. Protein quantification was carried out using the *DC*™ Protein Assay (BioRad) according to manufacturers’ instructions.

### Western Blot Analysis

The protein content of each sample was equalized to 0.55 mg ml^−1^, and 14 μl of each sample was separated by SDS-PAGE and transferred to a PVDF membrane. Western blot analysis was performed using anti-RsbL primary antibodies raised in rabbits (kindly provided by Jörgen Johannsson, Umeå University, Sweden) and mouse anti-rabbit secondary antibody (Santa Cruz Biotechnology). Blots were imaged using a chemiluminescent substrate (Amersham) on a LICOR Odyssey®Fc Imaging System (LI-COR Biosciences). Image Studio (LI-COR Biosciences) was used to process and analyze the image.

## Results

### Growth Phase and Temperature Influence Visible Light Resistance in *Listeria monocytogenes*

**Figure 1:**
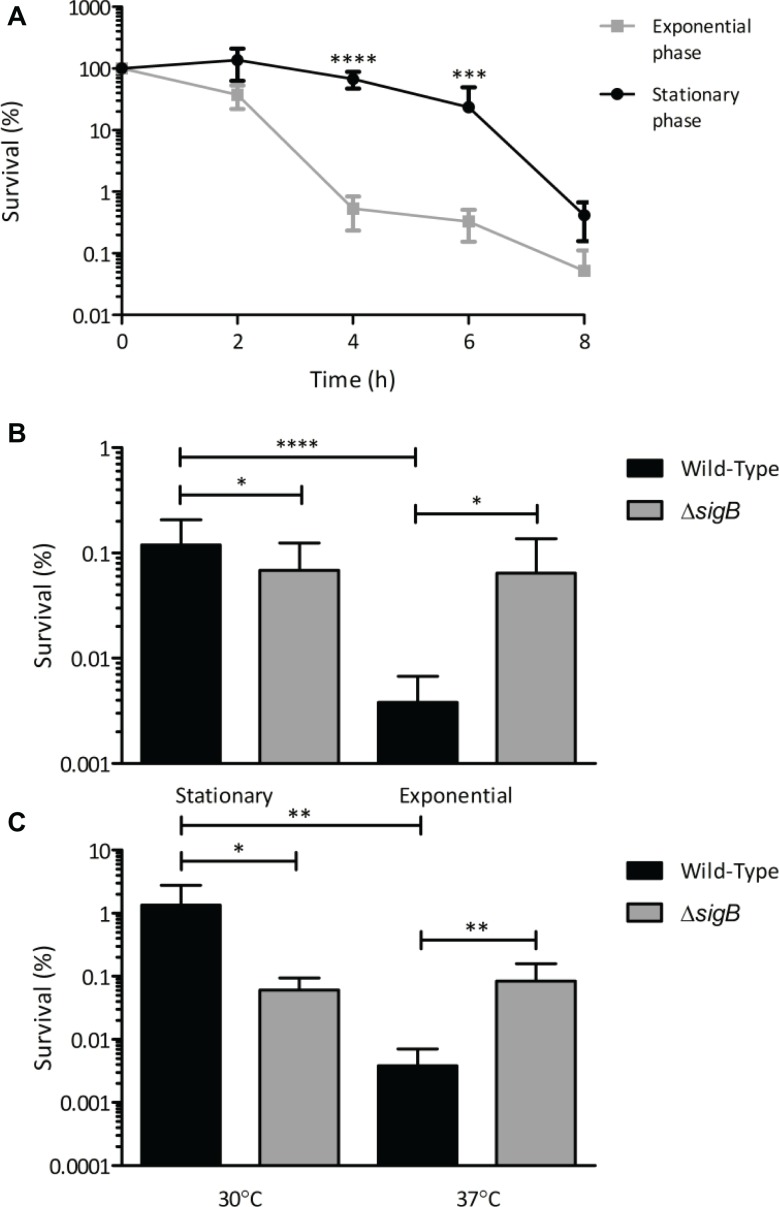
Sensitivity of *L. monocytogenes* to visible light is affected by growth phase and temperature. Cultures were grown to the desired growth phase in BHI broth at the appropriate temperature. Cells were centrifuged, washed once in PBS and resuspended in PBS to OD_600_ 1, and then exposed to 35 mW cm^−2^ 470 nm light for 8 h. Samples were taken at 0 h and at either 2 h intervals **(A)** or 6 h **(B,C)** and cfu/ml were calculated. Error bars represent SD from three technical replicates, plated in triplicate, of two independent replicates. Statistical significance was determined using either two-way ANOVA with Dunnett’s multiple comparisons test **(A)** or one-way ANOVA with Sidak’s multiple comparisons test **(B,C)**. (^*^p ≤ 0.05; ^**^p ≤ 0.01; ^***^p ≤ 0.001; ^****^p ≤ 0.0001).

In the present study, we examined the influence of growth phase on the resistance of *L. monocytogenes* to killing by visible light. EGDe wild-type cells were grown to either early exponential (OD ~ 0.2) or stationary phase at 37°C and exposed to 35 mW cm^−2^ 470 nm (blue) light over an 8 h period. After 4 and 6 h exposure to visible light, there was significantly (*p* ≤ 0.001) more killing of cells in the exponential phase of growth (100-fold decrease) compared to those in stationary phase (5-fold decrease) (Figure 1A). To determine the role of σ^B^ in this effect at 37°C, the survival of the wild-type and Δ*sigB* mutant strains at stationary and exponential phase were compared after 6 h of exposure to 35 mW cm^−2^ 470 nm (blue) light at 30°C. After 6 h, the wild-type exponential phase cells showed significantly (*p* ≤ 0.001) less survivors (0.005% survival) compared to the wild-type stationary phase cells (0.1% survival). In contrast, the Δ*sigB* mutant showed no significant (*p* ≥ 0.05) differences in the number of survivors between the two growth phases (0.08% survival) (Figure 1B). Finally, we examined the effect of growth temperature on the resistance of *L. monocytogenes* to visible light. Cells were grown to exponential phase (OD ~ 0.2) at 30 and 37°C and exposed to 35 mW cm^2^ 470 nm light. After 6 h, the wild-type cells grown at 30°C had a significantly (*p* ≤ 0.01) higher number of survivors (1% survival), compared to those grown at 37°C (0.008% survival) (Figure 1C). At 30°C, the Δ*sigB* mutant had a significantly (*p* ≤ 0.05) reduced survival (0.1% survival) compared to the wild-type (1% survival). However, unexpectedly at 37°C, the Δ*sigB* mutant (0.11% survival) had a significantly (*p* ≤ 0.01) greater survival than the wild-type (Figure 1C). These results suggest that both growth phase and growth temperature influence the resistance of *L. monocytogenes* to visible light, with stationary phase cells and cells grown at 30°C showing less sensitivity. The results also suggest that the role of σ^B^ in light resistance may vary as a function of growth phase and temperature.

### Activation of σ^B^ by Blue Light Is Temperature-Dependent

**Figure 2:**
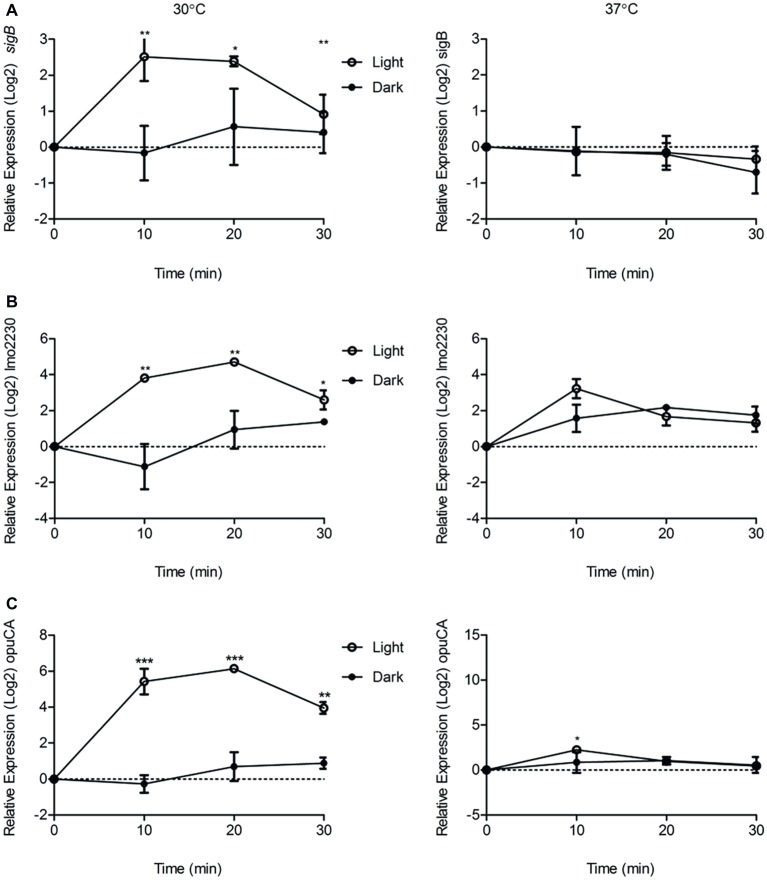
Exposure to visible light significantly increases the transcription of *sigB*, *lmo2230*, and *opuCA* compared to the dark control at 30°C but not 37°C. Cells were grown to OD ~ 0.2 at 30 or 37°C and exposed to 0.6 mW cm^−2^ 470 nm light for 30 min at the same temperature, with RNA samples collected at 10 min intervals. Relative transcription of *sigB* **(A)**, *lmo2230* **(B)**, and *opuCA* **(C)** was measured by RT-PCR. Error bars represent SD from two independent replicates. Statistical significance was determined by a paired *t*-test. (^*^p ≤ 0.05; ^****^p ≤ 0.0001).

As we had shown that the contribution of σ^B^ to surviving blue light stress in *L. monocytogenes* is dependent on the growth temperature, the influence of growth temperature on σ^B^-dependent gene transcription in the presence of sub-lethal blue light (0.6 mW cm^−2^) was investigated. The transcriptional response of *sigB* and two other σ^B^-dependent genes, *lmo2230* and *opuCA*, were measured following exposure to blue light at 30 and 37°C over a period of 30 min. The 30 min time frame was selected as Utratna et al. (2011) previously demonstrated a peak in SigB activity in response to osmotic stress after 15 min exposure. Cells cultured at 30°C showed a significant (*p* ≤ 0.05) increase in the transcription of all three genes in the presence of blue light compared to the dark control over a 30 min period (Figure 2). However, in cells cultured at 37°C, there were no significant differences in the levels of transcription of either *sigB* or *lmo2230* in the presence or absence of blue light (Figure 2). A small but significant (*p* ≤ 0.0001) difference was seen in the transcription level of *opuCA* after 10 min, but this difference was not seen after 20 or 30 min (Figure 2C). Taken together, the data suggest that the activation of σ^B^ by blue light is temperature-dependent, with a greater increase in activity demonstrated at 30°C than at 37°C, a result that may help to explain the differences in sensitivity to light at these temperatures (Figure 2).

### The Expression of *rsbL* Is Not Temperature-Dependent

**Figure 3:**
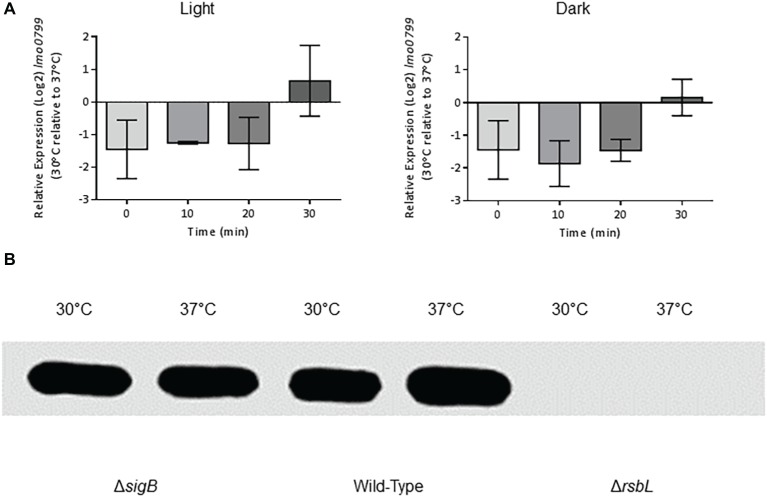
The expression of *lmo0799* is unaffected by temperature. **(A)** Cells were grown to OD ~ 0.2 at 30 or 37°C and exposed to 0.6 mW cm^−2^ 470 nm light for 30 min at the same temperature, with RNA samples collected at 10 min intervals. Relative transcription of *lmo0799* to T0 at each temperature was measured by RT-PCR. Error bars represent SD from two independent replicates. Statistical significance was determined by two-way ANOVA with Dunnett’s multiple comparisons test. **(B)** Protein extracts from stationary phase cultures of the wild-type, Δ*sigB* and Δ*lmo0799* strains grown at 30 or 37°C in darkness were standardized to 0.55 mg ml^−1^ and separated *via* SDS-PAGE. The levels of Lmo0799 in cultures were determined by western blot with polyclonal anti-Lmo0799 antibodies, with the Δ*lmo0799* strain as a negative control. Image is representative of three independent replicates.

One possible explanation for the apparent lack of light-mediated σ^B^ activation at 37°C was that the light sensor protein RsbL might not be expressed at this temperature. To determine whether the reduced activation of σ^B^ by blue light at 37°C compared to 30°C was due to the reduced transcription of *rsbL* at 37°C compared to 30°C, the difference in *rsbL* transcription at 30°C compared to 37°C was measured by RT-PCR over a 30 min period. No significant (*p* ≥ 0.05) differences were detected in the transcription of *rsbL* in cells cultured at 30 or 37°C (Figure 3A). In addition, we measured the changes in *rsbL* transcription in the presence and absence of blue light in cells cultured at 30 and 37°C. Again, no significant (*p* ≥ 0.05) differences were detected (Figure 3A). To confirm that the levels of the RsbL sensor protein are not affected by growth temperature, we quantified the levels of RsbL *via* Western blotting using anti-RsbL antibodies. The levels of RsbL were found to be unaffected by growth temperature (Figure 3B). Together, these results suggest that *rsbL* is not affected by either growth temperature at the transcriptional or translational levels.

### RT-PCR Confirms the Requirement for Cys56 to Alter the Transcription of Genes Under the Control of σ^B^

**Figure 4:**
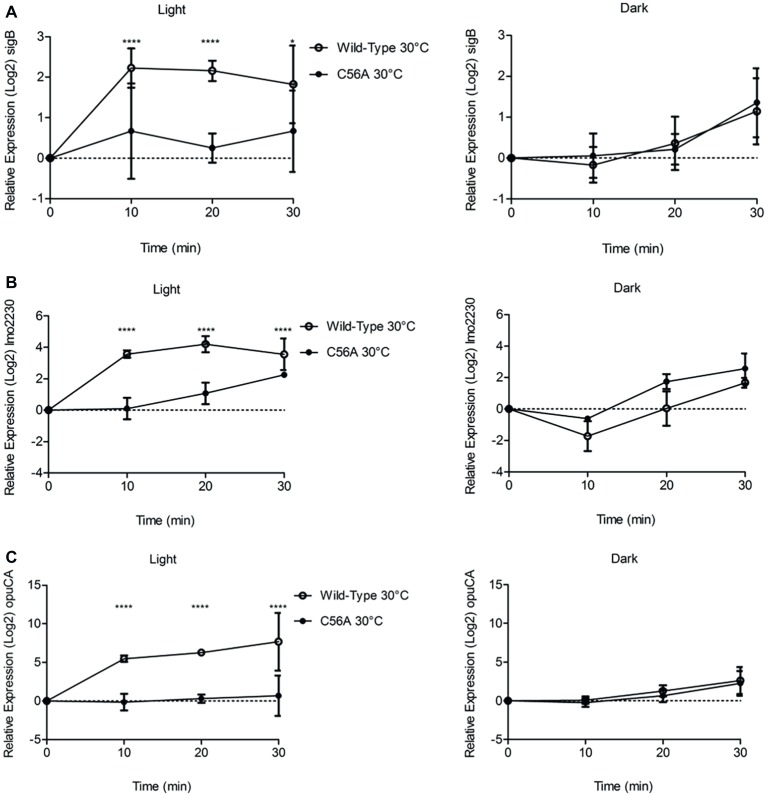
Increases in the transcription of *sigB*, *lmo2230*, and *opuCA* in the presence of visible light at 30°C is dependent upon Cys56 in Lmo0799. Exponentially growing cells were exposed to 0.6 mW cm^−2^ 470 nm light for 30 min at 30 or 37°C, with RNA samples collected at 10 min intervals. Relative transcription of *sigB* **(A)**, *lmo2230* **(B)**, and *opuCA* **(C)** was measured by RT-PCR. Error bars represent SD from three independent replicates. Statistical significance was determined by two-way ANOVA with Dunnett’s multiple comparisons test (****p ≤ 0.0001).

Next, we utilized RT-PCR to investigate the requirement for the conserved cysteine residue at position 56 in RsbL, previously identified as required for blue light sensing (O’Donoghue et al., 2016) in transducing the blue light signal to σ^B^, and consequently activation of the transcription of three σ^B^-dependent genes, *sigB*, *lmo2230*, and *opuCA*. The changes in transcription of all three genes were measured over a 30 min period at 10 min intervals following exposure to blue light at 30°C. The changes in transcription of *sigB* and the two σ^B^-dependent genes, *lmo2230* and *opuCA*, in the presence of blue light were abolished in the absence of RsbL Cys56 (Figure 4). For all three genes, the isogenic parental strain showed statistically significant (*p* ≤ 0.05) increased transcription compared to the C56A mutant strain. RT-PCR showed the wild-type strain having 2-, 4-, and 5-log_2_ fold changes in *sigB*, *lmo2230*, and *opuCA*, respectively, compared to the dark control. Transcription of these genes was also compared between the two strains in the absence of blue light, and no statistically significant (*p* ≥ 0.05) differences were detected (Figure 4). Thus, these results suggest that C56 is required for the activation of σ^B^ by blue light, though its absence does not affect σ^B^ in the absence of blue light.

### The Exposure of *Listeria monocytogenes* to Visible Light Significantly Alters the Transcription of 603 Genes

**Figure 5:**
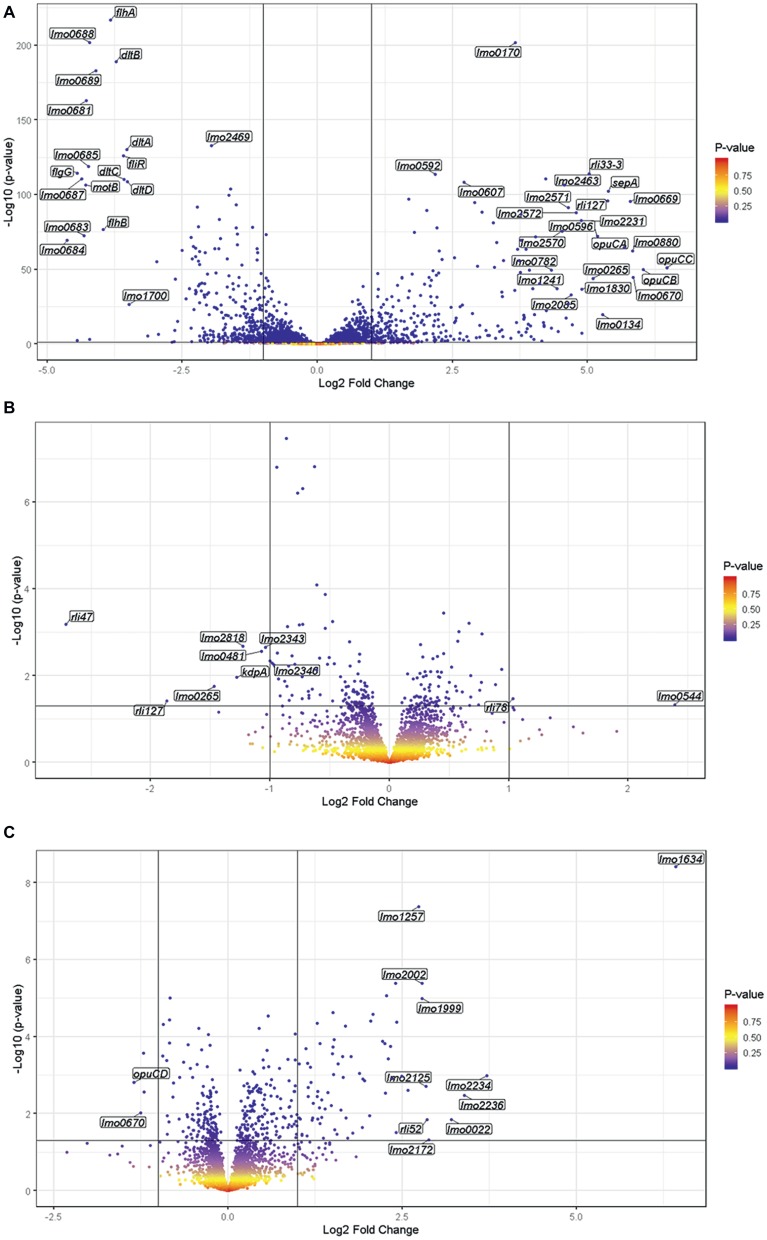
The exposure of *L. monocytogenes* to visible light significantly alters the transcription of 603 genes. RNA was sampled from exponentially growing cells exposed to either 0.6 mW cm^−2^ 470 nm light or darkness for 20 min at 30°C. Gene transcription was measured by RNA seq. and differential gene expression and the values of *p* were determined using DESeq2. The wild-type **(A)**, Δ*sigB* mutant **(B)**, and *rsbL*-C56A mutant **(C)** were included in this experiment.

To investigate the global transcriptional response to blue light and to determine possible mechanisms that account for the increased sensitivity of the Δ*sigB* mutant to visible light compared to the wild-type and *rsbL*-C56A strains, whole transcriptome analysis was carried out for all three strains in both the absence (dark) and presence of 0.6 mW cm^2^ blue (470 nm) light for 20 min at 30°C. The 20 min time interval was selected as RT-PCR data suggested that this exposure time resulted in the highest levels of SigB activity of the exposure times tested. This sub-lethal dose of light was chosen in line with previous work conducted by Ondrusch and Kreft (2011). In the presence of blue light in the wild-type strain, the transcription of 603 genes was significantly (*p* ≤ 0.05; log_2_ fold change ≥2) altered compared to the dark control (Figure 5A). Of these 603 genes, 308 were up-regulated and 295 were down-regulated, with *opuCC* showing the largest increase in transcription (6.47 log_2_ fold change) and *lmo0684* showing the greatest decrease in transcription (−4.63 log_2_ fold change). An analysis of the functional categories affected by blue light revealed that a large proportion of the downregulated genes are involved in cell envelope and cellular processes. When these were further divided into sub-categories, the mobility and chemotaxis sub-category was highly over-represented; 23.64% of significantly downregulated genes compared to 5.02% of the whole genome. Genes upregulated by blue light were distributed evenly across all functional categories and many belonged to the σ^B^ regulon. Taken together, these results indicate that exposure to low levels of visible light leads to significant changes in gene transcription, with a large proportion of the negatively affected genes being involved in mobility and chemotaxis.

To specifically investigate the role played by σ^B^ in the changes in gene expression detected in response to visible light, whole transcriptome analysis was also conducted using the Δ*sigB* mutant. In the absence of σ^B^, only 10 genes were found to be significantly altered in response to visible light exposure compared to the dark control, with 2 up-regulated and 7 down-regulated (Figure 5B). In contrast to the wild-type, the Δ*sigB* mutant significantly increased the transcription of *rli78* and *lmo0544* and significantly decreased the transcription of *lmo0481* and *lmo2818.* These genes were distributed across several functional categories (Table 3), with three being identified as transporters (*lmo0544*, *lmo2818*, and *kdpA*). Overall, these results suggest that the transcriptional response to blue light in *L. monocytogenes* is largely dependent on the stress inducible sigma factor σ^B^, potentially accounting for the light-sensitive phenotype observed in the mutant lacking this sigma factor (Figure 1).

**Table 3:** Genes with significantly altered gene expression in the presence of visible light, in a Δ*sigB* mutant.

Gene name	Log_2_ fold change	Functional category	RAST_product
*lmo0544*	2.39	Transport/binding proteins and lipoproteins	PTS system, glucitol/sorbitol-specific IIC component
*RatA-1 (rli78)*	1.03	sRNA	Unknown
*lmo2346*	−1.00	From other organisms	ThiJ/PfpI family protein
*lmo2343*	−1.04	Detoxification	Coenzyme F420-dependent N5,N10-methylene tetrahydromethanopterin reductase and related flavin-dependent oxidoreductases
*lmo0481*	−1.07	From other organisms	Putative antigen
*lmo2818*	−1.23	Transport/binding proteins and lipoproteins	Putative transporter
*kdpA*	−1.28	Transport/binding proteins and lipoproteins	Potassium-transporting ATPase A chain
*lmo0265*	−1.47	Metabolism of amino acids and related molecules	Acetylornithine deacetylase
*rli127*	−1.87	sRNA	Unknown
*sbrE (rli47)*	−2.71	sRNA	Unknown

### The Light Sensor RsbL Contributes to Light-Dependent Changes in Gene Expression

To investigate the contribution of the light sensor RsbL to the changes in gene expression produced in response to blue light, a whole transcriptome analysis was carried out using the light-blind *rsbL-*C56A mutant in response to blue light. In this strain, which is unable to sense visible light (O’Donoghue et al., 2016), the transcription of 77 genes was altered by blue light (Figure 5C), compared to 603 in the wild-type under the same conditions. Of the genes with significantly altered transcription, 32 also showed significantly altered transcription in the wild-type, while 45 were uniquely affected in the *rsbL*-C56A mutant strain. Of the genes whose transcription changed significantly in response to visible light exposure, there is an over representation of genes involved in intermediary metabolism and information pathways, and an under representation of genes involved in cell envelope and cellular processes, other functions, similar to unknown proteins, and sRNAs. Overall, these data suggest that the blue light sensor RsbL makes a significant contribution to the transcriptional response of *L. monocytogenes* to blue light, but further reveals that some σ^B^-dependent gene expression can still occur in response to light independently of the known light sensing mechanism in RsbL. This residual capacity to respond to light might help explain the difference in the light sensitivity between the Δ*sigB* mutant (sensitive) and the *rsbL*-C56A mutant (tolerant).

**Figure 6:**
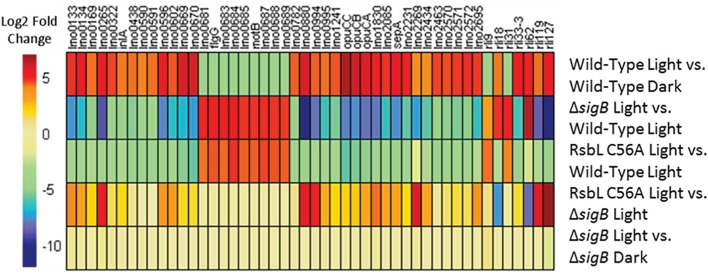
SigB is required for the altered transcription of 98% of genes showing a greater than 16-fold change in transcription in response to visible light. Genes showing a greater than 16-fold change in transcription in the wild-type in response to visible light were selected. All genes except for *rli18* and *rli62* require SigB to significantly alter their transcription in response to visible light.

To investigate whether σ^B^-dependent changes in gene transcription were occurring in the *rsbL*-C56A mutant strain in response to light, a heat map showing those genes that were most affected by light in the wild-type was generated and compared to the changes observed in the other conditions. Almost all genes showing a change in transcription >log_2_ = 4 in response to light in the wild-type were unaffected by light in the Δ*sigB* mutant. This is clearly seen on the heat map when the Δ*sigB* mutant and wild-type were compared to each other after light exposure (Figure 6, *ΔsigB* light vs. wild-type light); this comparison reveals an inverse relationship in the direction of the effect compared to effect of light on the wild-type (Figure 6, wild-type light vs. wild-type dark). There were two exceptions to this trend; *rli18* and *rli62*, both of which encode sRNAs, were induced by light independently of σ^B^ (Figure 6, *ΔsigB* light vs. wild-type light). The *rsbL*-C56A mutant strain also showed this inverse relationship, with almost all of the light-affected genes showing the opposite effect in this strain compared to the wild-type (Figure 6, *rsbL*-C56A light vs. wild-type light). Interestingly, a comparison of the *rsbL*-C56A mutant to the Δ*sigB* mutant in the presence of light revealed that the *rsbL*-C56A mutant retained an altered pattern of gene expression in response to light, albeit not to the same extent as the wild-type (Figure 6, *rsbL*-C56A light vs. Δ*sigB* light). This suggests that although the response to light is attenuated in a mutant lacking, a functional RsbL light sensor, σ^B^-dependent changes in gene transcription still occur albeit to a reduced extent.

## Discussion

### Influence of Growth Phase and Temperature on Sensitivity to Visible Light

Growth phase and temperature were shown here to alter the sensitivity of *L. monocytogenes* to visible light. In addition, the role of σ^B^ in protecting against visible light was found to be temperature-dependent. The influence of growth phase on the sensitivity of *L. monocytogenes* to environmental stresses has previously been reported for acid stress (Davis et al., 1996), heat (Lou and Yousef, 1997), and hydrostatic pressure (Mackey et al., 1995; Saucedo-Reyes et al., 2009), with cells in the stationary phase of growth to be more resistant to these stresses than those in the exponential phase. Utratna et al. (2011) demonstrated a proportional increase in σ^B^ activation as growth phase increased in response to osmotic stress. As we have previously identified a role for σ^B^ in visible light tolerance (O’Donoghue et al., 2016), we hypothesize that the increased resistance of *L. monocytogenes* to visible light in stationary phase is due to increased σ^B^ activity in stationary phase. The data in this study suggest that this is the case, as the Δ*sigB* mutant showed no alteration in resistance to visible light in response to changes in growth phase (Figure 1B).

The change in growth temperature from 30 to 37°C significantly reduced the survival of the wild-type 100-fold, but the Δ*sigB* mutant was unaffected. While this result was unexpected, the presence of σ^B^ has been associated with increased sensitivity of *L. monocytogenes* EGDe to hydrogen peroxide at 37°C but not at 30°C (Boura et al., 2016). As the mechanism by which visible light kills *L. monocytogenes* is thought to involve reactive oxygen species (O’Donoghue et al., 2016), it seems possible that the role of σ^B^ could be similar during exposure to visible light or hydrogen peroxide. In agreement with our study, Boura et al. (2016) also demonstrated an increased sensitivity of the wild-type to hydrogen peroxide at 37°C compared to 30°C. A previous study has shown that the Δ*sigB* mutant has increased motility at 37°C compared to the wild-type (Raengpradub et al., 2008), potentially due to inhibited expression of *mogR* (Toledo-Arana et al., 2009).

Considering the deleterious effect of σ^B^ at 37°C in resistance to visible light (Figure 1B), we hypothesized that there may be a difference in the activity of σ^B^ between the two temperatures in response to visible light exposure. This differential activation of σ^B^ by light at 30 and 37°C was not due to a temperature-dependent change in the expression of *rsbL*, since both the transcription and translation of *rsbL* were unaffected by the growth temperature (Figure 3). One possibility is that the FMN cofactor required for blue light sensing might associate with the sensor protein RsbL in a temperature-dependent manner. In this regard, it is noteworthy that Chan et al. (2013) demonstrated a reduced retention of the FMN chromophore by RsbL as temperatures increase above 26°C. Although somewhat unexpected, the finding that σ^B^ activation by light is absent at 37°C does potentially make physiological sense. When *L. monocytogenes* is exposed to 37°C, it is most likely to be within a mammalian host, an environment where light exposure is essentially absent. There would be no selective pressure to retain the capacity to sense light at this temperature. As σ^B^ is known to be active in the host and *inlA* expression is under the control of σ^B^ in the gastrointestinal tract (Toledo-Arana et al., 2009), it is possible that σ^B^ is activated by the presence of acid and bile in this environment as opposed to temperature. At typical environmental temperatures outside the host (30°C and below), the capacity to sense light and mount an appropriate protective response would be restored, thereby facilitating survival of any solar irradiance encountered.

### Visible Light Exposure Represses Transcription of *Listeria monocytogenes* Motility Genes

To investigate the transcriptional response to blue light, transcriptomics was performed on *L. monocytogenes* wild-type cells cultured in the presence of low intensity visible light or darkness for 20 min. Compared to the dark control, visible light exposure altered the transcription of over 600 genes (Figure 5). Approximately half of these genes had previously been shown to be σ^B^-dependent, suggesting that transcription of the σ^B^ regulon is expressed in response to visible light. This result was not unexpected, as previous studies have shown that exposure to visible light increases the transcription of several σ^B^-dependent genes (Ondrusch and Kreft, 2011; Tiensuu et al., 2013). Interestingly, a previous study by Uesugi et al. (2016) did not detect significant upregulation of any genes after the exposure of *L. monocytogenes* to pulsed light of wavelength greater than 400 nm. The difference between the results of the two studies may be due to the increased dose of light in the current study (42 J/m^2^) compared to the previous study (0.033 J/m^2^), suggesting that the effect of visible light exposure on gene transcription could be dose-dependent. Moreover, the study by Uesugi et al. (2016) used a pulsed light filter to remove UV wavelengths, therefore exposing the bacteria to light across the entire visible spectrum, rather than just 470 nm as in the current study.

An analysis of the functional categories of genes affected by blue light identified a large proportion of downregulated genes that were involved in cell motility and chemotaxis. Previous studies have shown that visible light exposure inhibits cell motility (Ondrusch and Kreft, 2011; Tiensuu et al., 2013; O’Donoghue et al., 2016), so a downregulation of genes involved in motility offers an explanation of the molecular mechanism for this response. A σ^B^ promoter is located upstream from MogR, a transcriptional repressor of motility genes, (Toledo-Arana et al., 2009), so the downregulation of motility genes in the presence of visible light is likely to be due to increased σ^B^ activity leading to increased expression of MogR, and therefore increased repression of motility gene transcription. The decrease in motility gene transcription mirrors the results of the previous study by Uesugi et al. (2016), suggesting that a lower dose is required to trigger this response than is required to increase gene transcription. While the physiological advantage for this phenotype is unknown, the inhibition of motility by visible light is not specific to *L. monocytogenes* and has been shown in both *E. coli* and *Salmonella typhimurium* (Taylor and Koshland, 1975). Indeed, it would seem that a lack of motility in the presence of visible light could be deleterious to the bacterium as it would be unable to migrate to a dark environment; however, decreased motility may be an energy-saving mechanism. The initiation of the general stress response is an energy intensive process for the cell, as is motility, so the inhibition of motility by the general stress response may enable the cell to conserve energy for use in alternative protective and homeostatic processes.

In the absence of σ^B^, alterations in transcription were limited to 10 genes, emphasizing the dominance of σ^B^ in the response to visible light. As expected, no genes involved in cell motility showed significant changes in transcription in response to visible light in the Δ*sigB* background, confirming that changes in motility gene transcription are σ^B^-dependent. In previous studies investigating the effects of visible light on cell motility, the Δ*sigB* mutant has shown no changes in motility in response to visible light (Ondrusch and Kreft, 2011; Tiensuu et al., 2013; O’Donoghue et al., 2016). The current study provides molecular evidence that σ^B^ is required for the inhibition of motility in response to visible light. The reduction in the number of significant changes in gene transcription from 600 to 10 genes in response to visible light exposure in the absence of σ^B^ likely explains the increased sensitivity of the Δ*sigB* mutant to kill by visible light. It seems likely that genes belonging to the σ^B^ regulon contribute to protection and repair functions that help to mitigate the damaging effects of blue light. The presence of three differentially regulated transporters identified as having significantly altered expression during blue light exposure in the Δ*sigB* mutant, suggesting the possibility that the accumulation of some small molecules might either increase or decrease the sensitivity of *L. monocytogenes* to blue light. Future experiments investigating the impact of the removal of these transporters from the genome may be useful in determining both the role of these transporters in the response of *L. monocytogenes* to blue light and also in identifying the molecules transported by the transporters.

As with the Δ*sigB* mutant, the *rsbL*-C56A mutant showed a reduction in the number of genes showing significant changes in transcription; just 77 genes were affected in this strain in response to visible light exposure compared to 600 in the wild-type. When compared to the wild-type, no genes showing significant up or down-regulation of transcription in the *rsbL*-C56A mutant mirrored the changes seen in the isogenic parental strain after exposure to visible light. However, when the *rsbL*-C56A mutant was compared to the Δ*sigB* mutant after exposure to visible light, 75% of genes that showed a significant change in gene transcription in response to visible light showed the same trend in transcriptional change as the wild-type in response to visible light, albeit to a lesser extent (Figure 6). Included in these genes showing an intermediate change in transcription in the *rsbL*-C56A mutant were the highly σ^B^-dependent genes *opuCA* and *lmo2230*, suggesting that *L. monocytogenes* is able to activate σ^B^ in response to visible light *via* an alternative mechanism that is independent of the light sensing capacity of RsbL. This intermediate effect may offer an explanation for the unexpected difference in resistance of the *rsbL*-C56A mutant compared to the Δ*sigB* mutant when challenged with a lethal dose of visible light exposure. This result also suggests that *L. monocytogenes* is able to sense and respond to secondary stresses associated with visible light exposure. This is not the first time that a degree of redundancy has been associated with the different RsbR paralogues, the sensory proteins of the stressosome. A study on the responses of *B. subtilis* to ethanol stress utilizing mutants lacking all bar one RsbR paralogue found that all of the mutants were able to respond to ethanol stress, but the pattern and amplitude to which they responded to the stress varied (Cabeen et al., 2017). However, investigations into the activation of σ^B^ by blue light *via* YtvA in *B. subtilis* suggest that the deletion of *ytvA* abolishes σ^B^ activation by blue light (Ávila-Pérez et al., 2006).

Interestingly, there were no significant differences in the transcription of motility genes, including the transcriptional repressor MogR, between the *rsbL*-C56A and Δ*sigB* mutants in response to visible light. This finding provides a possible explanation for the lack of motility repression in the *rsbL*-C56A mutant in response to visible light described previously (O’Donoghue et al., 2016), but also suggests that some σ^B^-dependent changes in gene transcription are dependent upon RsbL Cys56, but others can be activated *via* an alternative mechanism. This result, and also the previous finding by Uesugi et al. (2016) that motility can be repressed by a much lower dose of light than that required to increase the transcription of other σ^B^-dependent genes, suggests that there may be a more refined level of regulation of the general stress response in *L. monocytogenes* than previously thought. Investigations into the activation of σ^B^ in response to cold stress identified transcriptional changes in σ^B^-dependent genes in a Δ*rsbV* mutant, suggesting that σ^B^ activation can occur independently of RsbV (Utratna et al., 2014). Alternatively, a study by Yee et al. (2015) demonstrated that, in the absence of the conserved cysteine residue, LOV domains are able to reduce the FMN to the neutral semiquinone (NSQ) state in the presence of light photons, which is able to modulate downstream signaling in a way that is equivalent to that of cysteine adduct formation This suggests that *L. monocytogenes* may still be able activate SigB in response to visible light when Cys56 has been mutated to Ala, offering a potential explanation for why the *rsbL*-C56A mutant does not have an increased sensitivity to visible light, and why the mutant is also able to partially activate SigB in response to visible light.

### Conclusions

In conclusion, this study has shown that blue light sensing in *L. monocytogenes* is temperature dependent and that the global transcriptional response is highly dependent on σ^B^. We have shown that the effect of temperature on light sensing is unlikely to be caused by temperature-dependent differences in *rsbL* transcription or translation. This study presents, to our knowledge, the first whole genome transcriptomic investigation into the response of *L. monocytogenes* to visible light in both the presence and absence of σ^B^ and RsbL Cys56. Through utilization of the *rsbL*-C56A and Δ*sigB* mutants, the study has helped to define the roles for these proteins in light sensing and resistance. In addition, our results provide evidence that the inhibition of motility by visible light is due to increased σ^B^ activity. Finally, the results of this study suggest that the σ^B^ regulon can be partially activated by blue light in a way that does not depend on the light sensing functions of RsbL. The finding that some σ^B^ regulon genes are unaffected in the *rsbL*-C56A mutant suggests that there may be a degree of selectivity to the general stress response to visible light.

## Data Availability Statement

The raw data supporting the conclusions of this manuscript will be made available by the authors, without undue reservation, to any qualified researcher. The raw and processed data of the RNA seq analysis has been uploaded to Gene Expression Omnibus. Accession number GSE130971.

## Author Contributions

AD conducted all experiments and data analysis. CO’B conceived the study. AD and CO’B contributed equally to the designing of experiments, writing, and editing of the manuscript. B-HL conducted RNA seq analysis and BR conducted RNA seq data analysis.

### Conflict of Interest

B-HL and BR were employed by company GenXPro.

The remaining authors declare that the research was conducted in the absence of any commercial or financial relationships that could be construed as a potential conflict of interest.
